# On the Machinability of an Al-63%SiC Metal Matrix Composite

**DOI:** 10.3390/ma13051186

**Published:** 2020-03-06

**Authors:** David Repeto, Severo Raul Fernández-Vidal, Pedro F. Mayuet, Jorge Salguero, Moisés Batista

**Affiliations:** Department of Mechanical Engineering & Industrial Design, Faculty of Engineering, University of Cadiz, Av. Universidad de Cádiz 10, E-11519 Puerto Real-Cádiz, Spain; raul.fernandez@uca.es (S.R.F.-V.); pedro.mayuet@uca.es (P.F.M.); jorge.salguero@uca.es (J.S.)

**Keywords:** metal matrix composite, Al-SiC, microstructure, machining

## Abstract

This paper presents a preliminary study of aluminium matrix composite materials during machining, with a special focus on their behavior under conventional processes. This work will expand the knowledge of these materials, which is considered to be strategic for some industrial sectors, such as the aeronautics, electronics, and automotive sectors. Finding a machining model will allow us to define the necessary parameters when applying the materials to industry. As a previous step of the material and its machining, an experimental state-of-the-art review has been carried out, revealing a lack of studies about the composition and material properties, processes, tools, and recommended parameters. The results obtained and reflected in this paper are as follows; SiC is present in metallic matrix composite (MMC) materials in a very wide variety of sizes. A metallographic study of the material confirms the high percentage of reinforcement and very high microhardness values registered. During the machining process, tools present a very high level of wear in a very short amount of time, where chips are generated and arcs are segmented, revealing the high microhardness of the material, which is given by its high concentration of SiC. The chip shape is the same among other materials with a similar microhardness, such as Ti or its alloys. The forces registered in the machining process are quite different from conventional alloys and are more similar to the values of harder alloys, which is also the case for chip generation. The results coincide, in part, with previous studies and also give new insight into the behavior of this material, which does not conform to the assumptions for standard metallic materials, where the hypothesis of Shaffer is not directly applicable. On the other hand, here, cutting forces do not behave in accordance with the traditional model. This paper will contribute to improve the knowledge of the Al-63%SiC MMC itself and the machining behavior.

## 1. Introduction

Metallic matrix composite (MMC) materials are the result of the combination of a metallic alloy matrix with reinforcement [1], giving rise to a unique combination of mechanical properties, which surpasses those of the individual components [2,3]. This improvement is given by the incorporation of a ductile metal matrix of reinforcements particles with a high strength and modulus of elasticity [4,5]. When compared to monolithic alloys, MMCs offer superior strength and stiffness values, lower weight and thermic expansion coefficients, and, in some cases, the ability to operate at high temperatures [6]. Because of its exceptional properties, and despite the complexities of its behavior throughout the machining process, there exists great industrial interest in MMC’s: several potential areas of application can be covered by these materials.

Each MMC is unique and its composition corresponds to a general criterion, which represents an added difficulty to its replicability of laboratory experiments, as well as subsequent industrial production.

The uneven distribution of the reinforcement accounts for the dispersion found by its behavior during the machining process, although some manufacturers that commercialize these products try to combine the matrix and straighteners in a particular manner in the lab in an attempt to control the process from the beginning.

A standard commercial material would guarantee similar results in successive experiments, although the use of a specific aluminium matrix is not currently well defined, and the choice appears to be conditioned by the matrix properties, as its behavior is improved by the addition of reinforcements. The microstructural analysis of the material revealed a distribution of reinforcements typical of an MMC, highlighting the high concentration (63%) and the differences in size, as well as the random distribution. Improving the quality and homogeneity of reinforcement particles would facilitate the MMC manufacturing process in terms of the required specifications. This is the part of the process where an exhaustive control is generally lacking in the literature.

Although the military industry has a specific interest in MMC applications (mainly for ballistics), the automotive sector has opened a broader field for these materials, motivated by the search of lighter vehicles. In the electronics sector, MMCs have direct applications in the manufacturing of microprocessors, due to their excellent heat transfer properties. Nowadays, MMCs are considered as strategic materials, forming part of the essential elements in many advanced technologies [7].

MMCs are designed according to their final purpose, based on the properties of the metal matrix (Al, Mg, Ti, Cu, etc.) and the particle reinforcement characteristics, varying by application and manufacturer [8]. Materials which are more resistant and possess a lower weight are required nowadays to replace lightweight alloys, and MMCs are in the best position to satisfy these requests. Adding ceramic reinforcements to an aluminium matrix helps to dramatically increase some properties, such as microhardness, thermal conductivity, and strength.

Al-based MMCs are usually manufactured with continuous (long fibers) or discontinuous reinforcements (short fibers, whiskers, wires, or irregular and spherical particles) [9,10].

Particulate-based metal matrix composites (PMMCs) are of particular interest, as they exhibit higher ductility and lower anisotropy than fiber-reinforced MMCs [11]. Because of this, reinforcements are distributed in a 4:1 ratio—in favor of the particles against short fibers [12]—and the selection of SiC as a reinforcement with an aluminium matrix is over 60% in this regard, because of its mechanical properties, characteristics, physical, and chemical properties, and low cost of production when compared with other reinforcements which could be used, such as Al_2_O_3_, with around 30% [13]. The rest of the various materials are not relevant in terms of their composition percentage, where 10%, which is quite dispersed, can be found for B_4_C, TiC, or hybrids of SiC and Al_2_O_3_ [14,15,16].

In terms of reinforcement quantity, [6,11,17,18,19,20,21,22,23], most of applications include at least 20% reinforcement, which can be considered as the highest ratio. Regarding reinforcement sizes, these are often between 3 and 65 μm; however, three-quarters of the studied sizes are ~20 μm [24].

The number of papers found regarding the machinability of Al-based MMCs with particulate reinforcement similar to the material studied here (over 60% Al) is small, and these papers are rare [25,26]. Consequently, it was decided to investigate this kind of material, which is the main objective of this paper.

## 2. Machining of Metal Matrix Composites

Quigley studied factors affecting the turning [27] of MMC 5083 AlSiC with 25% Al and found that the conventional and coated tool flank wear was very high; the surface finish was better than that which was expected theoretically, due to the tool morphology and material effect (BUE); and that tool wear leads to a loss in dimensional accuracy.

El-Gallab studied tool performance and workpiece surface integrity [11] when turning Duralcan F3S.20S AlSiC (A356/20% SiC) with Al_2_O_3_/TiC, TiN, and PCD tools, where all of the BUE and flank wear (VB) of all forms were measured. The cutting parameters (the speed of cut, feed, and depth of cut) play a key role in determining the amount of tool flank wear, as well as the size of the built-up edge. El-Gallab also noticed [19] that no research has yet been carried out to determine the effect of the cutting parameters on the workpiece surface integrity and sub-surface damage during the machining of an AlSiC particulate-based metal matrix composite, where cutting parameters have a significant effect on the form of the chips produced. In general, at low feed rates and small cut depths, the chips tended to be continuous, and this observation contradicts Monaghan’s results [28]; even though the parameters and materials were the same, there was a 5% SiC difference between them. A reduction in the surface roughness can be noticed with the increase in feed rate, and this effect is attributed to the reduction in the flank wear of the tool. This could be attributed to the stable built-up edge, which protects the tool from wear by abrasion. Manna investigated the machinability of an AlSiC MMC (A413/15% SiC) [29] and found that the lower built-up edge (BUE) is formed during the machining of AlSiC MMC at a high speed and low cut depth, while also observing a better surface finish at a high speed with a low feed rate and low cut depth.

Davim studied the optimization of cutting parameters based on orthogonal arrays [21], where turning A356/20SiCp-T6 with Poly Crystalline Diamond (PCD) tools showed that the cut speed is the main factor that affects tool wear and power consumption, whereas the feed rate is the main factor for surface roughness (Ra). Also, in 2007, Davim made a correlation between the chip compression ratio and shear plane angle or chip deformation during the turning of MMCs, showing that the shear angle decreases with the chip compression ratio. On the contrary, chip deformation has been shown to increase with the chip compression ratio [30]. The Merchant model gives, in general, an overestimation of the shear plane angle value in the cutting of Al-based MMCs.

Dabade investigated the effect of a change in size and volume fraction of reinforcement (Al/20–30 SiCp; 220–600) on the mechanism of chip formation by changing the processing conditions and tool geometries [31]. Here, the results were that at a lower cutting speed (40 m/min), thin needle-type flakes, as well as segmented chips are formed, whereas at higher cutting speeds (120 m/min), generally, semicontinuous, continuous, scrambled ribbon, and tubular helix chips are formed. The length of chip and the number of chip curls increases with the increase of feed rate at any given cutting speed and cut depth. The size and volume fraction of reinforcement significantly influences the chip formation mechanism.

One of the conclusions that can be drawn about the different manufacturing systems of MMCs is that they must evolve over time in terms of the method of incorporation of reinforcements into the matrix, so that the desired properties are established and maintained. Another concern, although to a lesser extent, is that the temperature reached during the manufacturing process should be kept as low as possible. The papers we have studied reflect the effort to manufacture a material that faithfully follows the design specifications of the experiment and does not raise concern about the scope of its cost. The objective in this study, differently to the objectives of other studies reported in the literature, is to move the manufacturing method into industry.

Although MMC production is relatively well adjusted to its final product shape, machining is unavoidable [32,33]. Nowadays, improvements of this process are the main focus of many research efforts [34]. Nevertheless, it is the combination of matrix and reinforcements that creates the enhanced properties of an MMC that hinder its machining, even for matrix-based aluminium alloys, which are considered to have a high machinability. Despite this, the machining of aluminium alloys is not exempt from difficulties, traditionally owing to the great capability of the material to adhere to tool edges and, above all, the shear angle [35].

The reinforcement itself represents an added difficulty, as the tool encounters a heterogeneous material throughout its path. If the reinforcement is harder than aluminium, as is the case, the machining becomes even more complex. Regarding MMCs with ceramic reinforcements (Al_2_O_3_ and SiC), the situation is further complicated by the abrasiveness of these materials, resulting in extremely damaging machining for traditional tools, shortening their service life drastically.

As the difficulty in machining MMCs increases because of the abrasion phenomenon described above, a greater force is required to pull the chips and the amount of energy required increases, which can greatly influence the total efficiency of the process [36]. The greatest obstacle encountered by MMCs in replacing monolithic alloys is the difficulties associated with their machining, as the tools required suffer from intense wear owing to the presence of the reinforcements, making it a highly inefficient process [6,37].

The shape, type, presentation, and concentration of the reinforcement greatly influence the wearing process. Similarly, a great dispersion between the responses in MMCs can be detected, as the characteristics of the particular composites integrating them can result in the manifestation of completely opposite properties.

In this paper, a study of the machinability of an Al-based MMC with a high concentration (63%) of SiC reinforcement has been performed. Machinability tests have been carried out by orthogonal machining (shaping) from some values of speed and deep of cut. The geometry of the chips obtained, as well as the characterization of cutting tool wear, have been studied by optical and scanning microscopy techniques; also, cutting forces are measured. This paper will allow to establish the basis of a parametrical analysis in regards MMC’s with high content SiC reinforcement or some other materials characterized by a similar behavior.

## 3. Experimental Procedure

The experimental procedure has been divided into two phases: The first one develops a microstructural characterization of the MMC to be machined, and this is necessary to know the distribution of the reinforcements. The second phase is focused towards the machining of this MMC. During and after the machining, the chip behavior has also been studied via high-speed video film and metallographical methods, including Stereoscopic Optical Microscopy (SOM) and Scanning Electron Microscopy (SEM) techniques.

### 3.1. Material Characterization

For this study, a commercial MMC sheet (ref. AlSiC-9) composed of an aluminium UNS A03562 (A356.2) matrix with 63% SiC reinforcement was used. The composition of the A365.2 Al alloy, obtained from the webpage Matweb, is shown in Table 1.

Even though this material has a commercial distribution, this does not mean that it is unnecessary to study it because it is not an alloy under international regulations. The sample was 180.17 mm wide and 136.89 mm long, with a thickness of 5.55 mm. The main usage of this plate was to support an electronic array for an insulated gate bipolar transistor (IGBT), provided by CPS Technologies Corp, Norton, MA, USA. The material properties are shown in Table 2.

**Figure 1:**
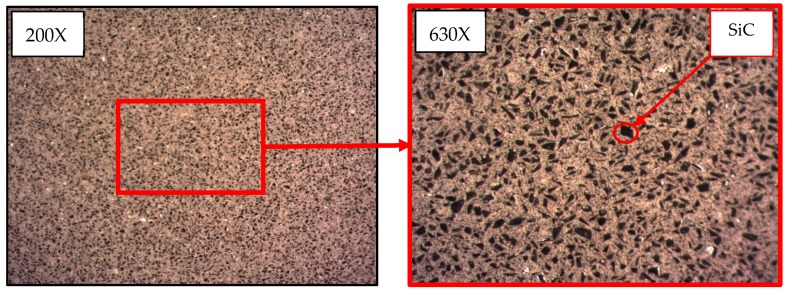
Metallography of the AlSiC MMC with 63% Al.

To characterize the microstructure, some samples were cut into an appropriate size and polished, prior to etching with Keller’s reactant. After, optical evaluation of the etched surface was carried out, as can be seen in Figure 1, using a Nikon SMZ 800 stereoscopic microscope and a Nikon Epiphot 200 metallographic microscope (Nikon Instruments, Amsterdam, Netherlands), both of which were equipped with digital high-resolution cameras.

Additionally, microhardness was measured with the HMV-2 (Shimadzu, Kioto, Japan) microhardness tester to evaluate the behavior in different phases of the matrix, as well as the properties of the reinforcement particles. Forces of 980.70 mN and 1.96 N were applied, using 100 and 200 grams, respectively, during 15 seconds, which corresponded to a Vickers Hardness (HV) value between 0.1 and 0.2, respectively, according to UNE-EN ISO 6507 [40].

### 3.2. Machining Process

**Figure 2:**
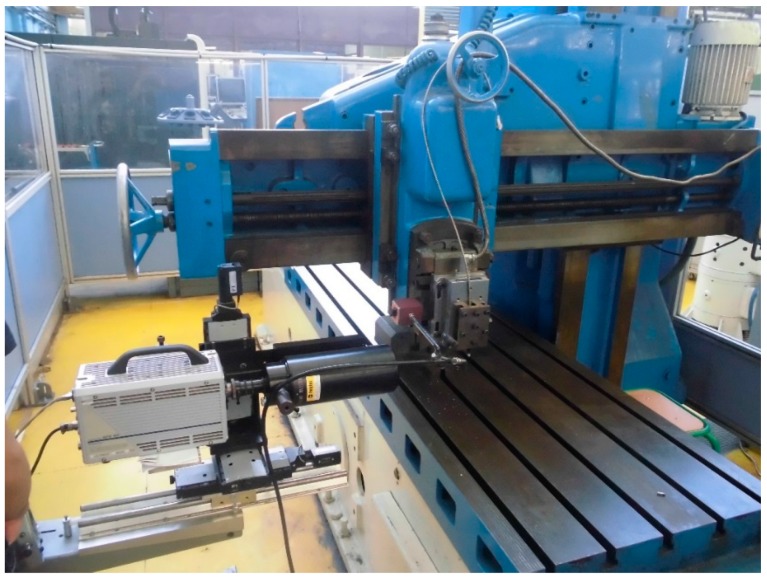
GSP 2108 R.20 shaping machine tool.

To study the machinability but avoid complex cutting geometries, it is typical to use machine tools with a rectilinear cutting movement, as this is the simplest machining model and it can be extrapolated to an oblique cutting model. Therefore, an orthogonal cutting configuration in a shaping machine (GSP 2108 R.20. GSP, Paris, France) was used, as shown in Figure 2.

All tests were performed in dry conditions, and a new cutting tool was used in each trial. The cutting parameters used were based on the combination of 4 cutting speeds and 2 cut depths selected from previous studies [41,42]. This is shown in Table 3.

Every test was carried out for at least 1 or 3 tool paths. The machining time ranged from 0.15 to 0.45 s, whereas the measured machining lengths were between 45 and 120 mm. The cutting forces were acquired by a Kistler 9257B dynamometer (Kistler Instruments, Winterthur, Switzerland), while the cutting process was filmed at high speed with a Photron APX RS (Photron, San Diego, CA, USA) camera.

**Figure 3:**
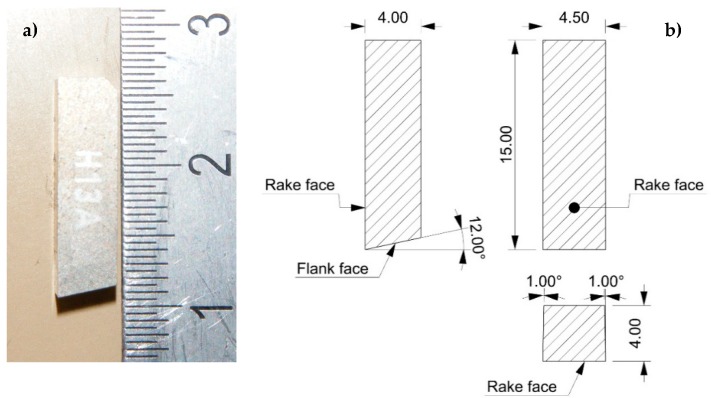
Blank cutting tool. (**a**) H13A from Sandvik. (**b**) Cutting geometry.

A WC/6%Co “blank” cutting-tool from Sandvik (ref. H13A, without coating) was selected, with a rake angle of 0° and a clearance angle of 12°, as shown in Figure 3.

**Figure 4:**
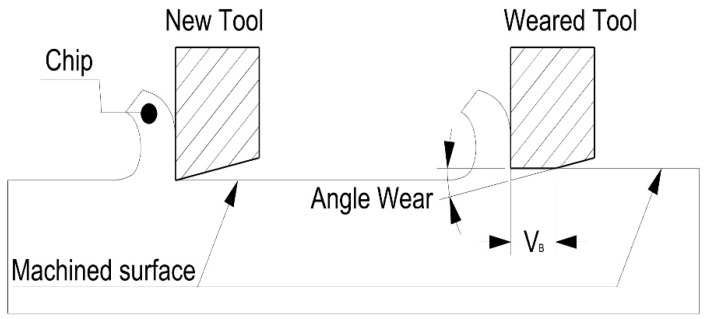
Orthogonal cutting configuration and evaluation zone for flank wear.

Chip morphology was analyzed according to ISO 3685:1993 (annex G). This is also a useful indicator of tool life. Angle wear can be observed by observing the tool wear via its initial profile, as shown in Figure 4.

Further evaluation of the material, chips, and tools was performed with a Quanta 200 scanning electron microscope (FEI, Hillsboro, OR, USA) equipped with a Phoenix Energy dispersive X-ray spectroscopy (EDX) system (EDAX, Mahwah, NJ, USA).

## 4. Results

### 4.1. Introduction

The manufacturing process of the MMC sheet was based on the method of infiltration by pressing the aluminium alloy over a preform of SiC. Infiltration was made in a cast created to give the final shape to the sheets, and its design corresponds to the net shaping criteria and techniques. Because of the manufacturing process, a skin of aluminium is formed over the sheet surface, which can reach a thickness of 0.08 mm and would need to be removed to find the “pure” MMC.

**Figure 5:**
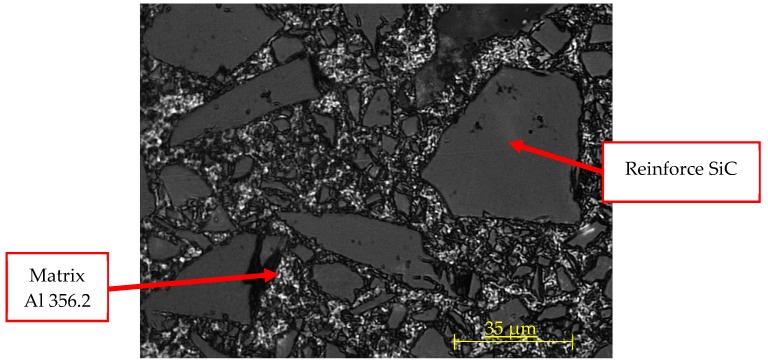
Stereoscopic Optical Microscopy (SOM) image at 40× magnification of the aluminium MMC with SiC reinforcements.

The images captured and shown above, Figure 5; allow the observation of the irregular dispersion of the reinforcement over the MMC surface, and also, its different sizes. Reinforcement shows two singular characteristics, namely, irregular shapes and a range of different sizes. The uneven distribution of the reinforcement sizes has been reported previously [6,43].

Using the picture processing software Image J, the areas occupied by each reinforcement were measured in four different places within the sheet. For this, a minimum-area size filter has been used for the initial pictures, so that smaller sizes are not considered, thereby eliminating the noise effect. The number of detectable size reinforcements in each of the four samples analyzed varies, almost reaching 12.50% for the those of the same material and between very similar study areas, with the lack of homogeneity being around 10%. Depending on the relative position of the reinforcement, the properties of the material can differ significantly.

The percentage of area occupied by detectable sizes of the reinforcement varies between 43.16% and 55.13%. Nonetheless, these values do not overlap with the maximum or minimum reinforcement quantities. The size and quantity of reinforcements is scattered, so that the properties of the material might have some heterogeneity.

Table 4 shows the microhardness values taken in different areas and samples of material. Relevant parameters are also included in the table. Registered values of microhardness, from highest to lowest, are shown. The highest value belongs to Sample 1 and was obtained in the reinforcement. The second value, from Sample 2, was taken in the matrix. Sample 3 had the second highest value, registered in the matrix-reinforcement interface. The last value, from Sample 4, was also taken from the matrix.

### 4.2. Machining Process

**Figure 6:**
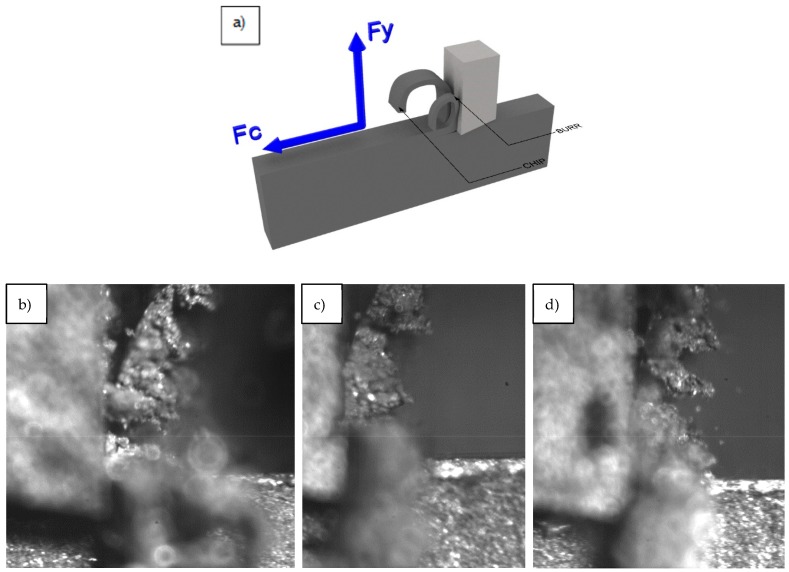
(**a**) Scheme of the tool position and images of the machining as obtained with high-speed film: (**b**) Vc = 20 m/min, one tool path, and d = 0.2 mm; (**c**) Vc = 30 m/min, three tool paths, and d = 0.2 mm; (**d**) Vc = 50 m/min, three tool paths, and d = 0.2 mm.

The high-speed film detected the formation of burrs from the very beginning of the machining process, which obstruct the visualization of the cutting zone. Furthermore, a great number of shoots were observed, and, although the surfacing of segmented and jagged chips in all cases is significant, this is an odd behavior for the matrix material at this particular cutting depth. Both of these properties provide some information about the brittleness of the chip (Figure 6).

The literature review on chip formation established that the machining process of aluminium MMCs with SiC reinforcements generates “sawtooth” chips because of the shape of cross section used to produce them [44]. However, a significant amount of the machined material was reduced to powder. This experiment corroborates [45] that reinforcements, which have a higher hardness, cause a reduction in MMC ductility, facilitating the appearance of these kinds of jagged chips. The tool rake angle of 0°, and even negative values, noticeably influences the formation of the chips.

Regarding the shoots, these present the phenomenon described [46]. The material cannot withstand the cutting tension and it breaks and is shot away once the chip has reached its maximum size. Some of the materials studied during the literature review do not belong to the metal matrix composites with ceramic reinforcement group. These are mostly very hard steels and some alloys employed in aeronautics, such as the titanium alloy Ti6Al4V or a nickel alloy known as Inconel 718. From the results obtained, we can observe a similar behavior in the MMC in terms of the chip formation mechanism and the type of chip resulting from the machining.

### 4.3. Chips Characterization

One of the characteristic aspects identified during the review of published papers was the cyclic variation of the shear angle along the machining process. This cyclic change of the shear angle in tandem with the chip formation angle can be verified [47]. In line with this, some fluctuations in the components of the cutting forces were observed while machining a nickel alloy, namely, Inconel 718 [48], although the resultant force did not change, owing to the cyclic variation of the chip segmentation (changes in the chip thickness from the shear angle). This phenomenon is still yet to be proven to occur in the case of aluminium MMC reinforced with SiC. The shootings observed during the film recording cannot allow us to confirm the adherence of the material to the cutting edge, although this is proven in later analysis.

After machining under the different experimental conditions scheduled, the morphology of the chips obtained were determined to be of the segmented type with a curved profile. Attending to International Standard Organization standard ISO 3685:1993, the chip formed during the cutting process has characteristics which are related to the work material, tool material, tool geometry, condition of the cutting edges, cutting edge position, and cutting data and conditions. According to annex G, these chips are of the 6.2 type (arched and shredded), which indicates that chip movement is produced towards the workpiece and in the direction of feed motion. However, during the cyclic chip-forming process, the shear angle is not simply a constant value, but changes cyclically as the cyclic chip formation takes place [48]. While machining ductile materials, chip formation is accompanied with very severe plastic deformation at the shear zone, where if a work material does not have enough ductility, this will result in deformation, which is limited by the crack initiation at the surface where no hydrostatic pressure exists [44]. As the reinforcement percentage is much higher in the case of the matrix, greater hardness is registered and the material is less ductile, resulting in the generation of a discontinuous chip because of high reinforcement concentration in the matrix [49].

Cutting forces in the machining of hard materials are, in spite of their hardness, not necessarily high because of the following two effects, namely, relatively small plastic deformation of the chip due to the crack formation mentioned above, and the relatively small area of tool-chip contact, which reduces the friction force [44].

The addition of SIC particle reinforcement into the aluminium matrix has caused a reduction in ductility and makes the material ideal for producing semicontinuous chips, which can be easily discarded after machining. This addition could be beneficial in terms of machinability; however, it creates some fluctuation in the force measurements [49]. Clarifying the mechanism and factors responsible for sawtooth chip formation and exploring the relationship between the formation of sawtooth chips and cutting force fluctuation is of great importance to the development of an effective cutting process monitoring strategy [48]. The face of the chip in contact with the tool is a smooth and shiny surface with crevices. This can be attributed to the lateral distortion forces induced by friction, which is a differentiating characteristic from traditional aluminium alloys [50].

The reverse or back side of the chip shows an irregular profile which is matt and discontinuous, allowing the shearing effects to show through and forming the characteristic sawtooth shape. Additionally, a burr area formed because of the fragility of the chip is observed in the high-speed film. It is possible to observe SiC particles near the chip surface, and these particles could damage the tool [51].

**Figure 7:**
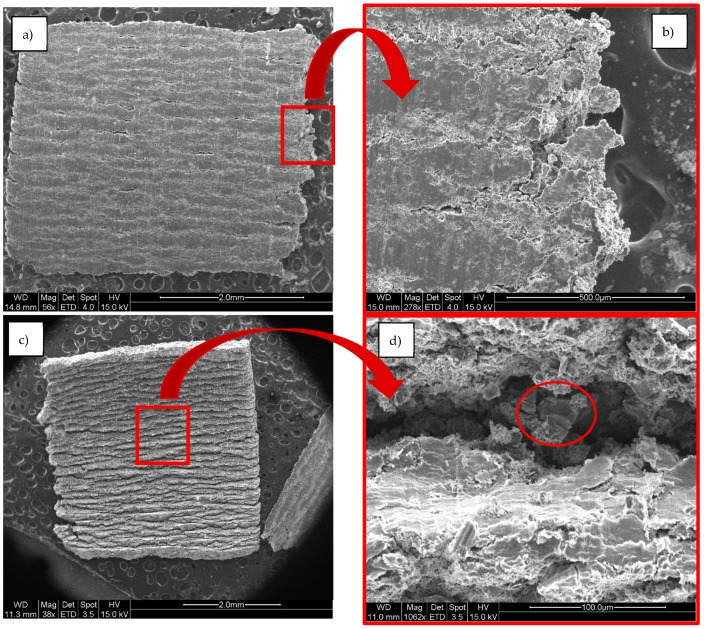
SEM images of MMC chip at Vc = 20 m/min, d = 0.2 mm, after three tool paths. (**a**) Front side. (**b**) Burrs at the front side. (**c**) Back side. (**d**) Reinforcement SiC particle in the back side.

These effects occurred under all of the studied conditions (Figure 7). The same shine and smoothness on the exterior size of the chips was observed [48]. This phenomenon is attributed to the high tensions which were created when in contact with the tool, and the shearing tensions occurring on the tool–chip interface. In the fracture zone of the chip, a lack of homogeneity on the fracture surface can be observed, and it can be concluded that said fracture cannot be considered ductile, but its appearance resembles that of a brittle fracture. This coincides with the increasing tenancy, related to the incorporation of reinforcements to an aluminium alloy, seen with the traditional characteristics of an MMC.

### 4.4. Tools Characterization

**Figure 8:**
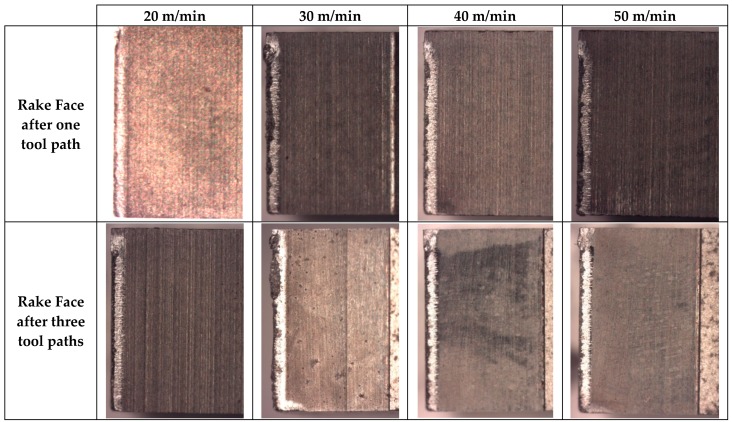
SOM images of tools used in machining test, with the rake face at 200X. Vc = 30 to 50 m/min, d = 0.2 mm.

In Figure 8, the rake faces of the tool are shown. The wear of the tool is significant in all cases, as can be seen by loss of tool material in the cutting edge and the material adhered in the rake face. Note that secondary adhesion is the most common mechanism related to the machining of aluminium alloys, and, in general, and it appears in this case with the MMC matrix. However, the SiC reinforcements should cause abrasion wear to the tool, and this favors material loss of the tool and provokes flank wear. Adhesion is presented almost consistently throughout the tool edge, creating an adhered layer or BUL (built-up layer). However, a clear relationship with the cutting parameters has not been revealed, although the BUL appears to increase as the machining time does, causing more damage to the tools after three tool paths.

The tool wear mechanism seems to be a mix between the dominant ones for each phase of the material. The secondary adhesion, distinctive for aluminium alloys (the matrix material), is boosted by high temperatures, as it is a thermomechanical mechanism. Besides, there is a loss of material produced by the abrasion that SiC reinforcements cause, which eliminates material from the tool edge and flank. This mechanism will also cause a rise in the temperature due to friction, which will favor secondary adhesion. The described mechanisms acting synergistically will result in the tool being rendered unusable after only very short machining times.

This behavior is found to be similar to those of different materials such as stacks, although it presents an added difficulty as the machining is not done in batches.

It is also remarkable that this mechanism seems to be dependent on cutting speed, as well as the machining time, and the effect increases at low velocities. This can be attributed to the change in behavior produced by friction, as an increase in friction is paired with greater abrasion, causing plasticity within the matrix, which results in an increase in adhesion, and thus the synergetic effect.

**Figure 9:**
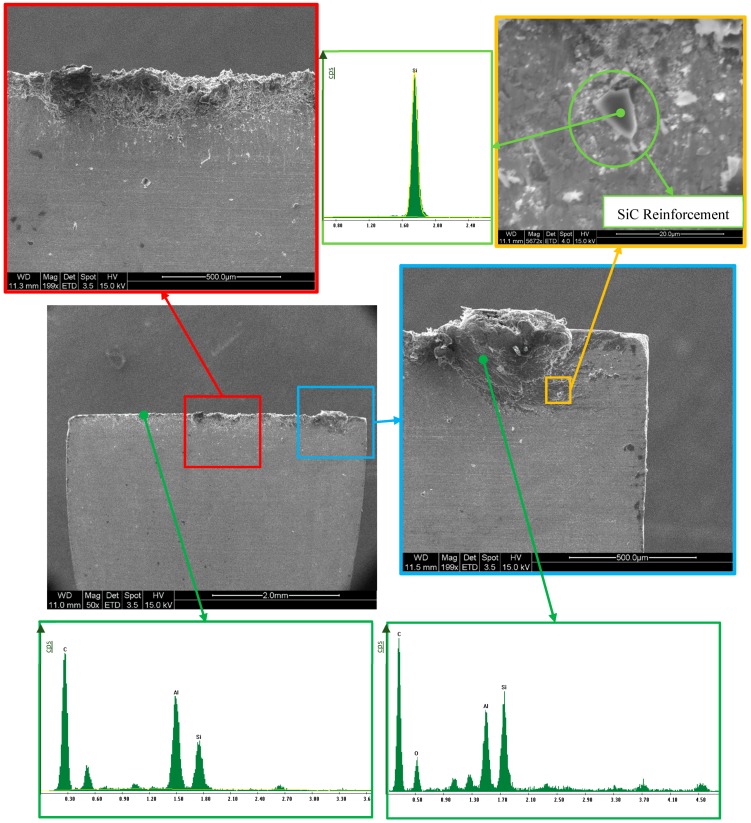
SEM/EDS analysis of the rake face of the cutting tool (Vc = 50 m/min, d = 0.2 mm, and one tool path). *Y*-axis in cps units. Two details of tool wear: figures on the **left** (red frame) are showing aluminium adhesion and figures on the **right** (blue and yellow frames) are showing SiC adhesion.

To quantify the adhesive wear, we quantified the length of contact between the tool and chip, referred to as h. After this analysis, it was noted that there is a fairly constant length, settling at 0.375 mm. This value allows quantification of the area damaged by secondary adhesion in the tool. Note that, according to the calculation made earlier, this value differs by up to 80% of the calculated value, meaning that the Shaffer model could not be applied for these materials, and that additional information is still needed to ensure the value of the shear angle. The tools were also analyzed with SEM/EDS techniques (Figure 9).

Analyzing the material adhered shows that it is material from the MMC; however, it features a different concentration of Al/SiC, depending the tool zone. In this form, zones rich in Al and others rich in SiC exist. This suggests that the reinforcement can be carried out at a high speed. This effect is in accordance with the particles shown by high-speed film and with the particles incorporated into the surface of the chip, where the effect can increase damage to the tool.

This fact could be considered secondary, because although it is of great importance from the point of view of the process, skipping particles of SiC with a high degree of abrasion and the projection itself can damage the tool, and this can also represent significant damage to equipment, meaning extreme care must be paid when machining these materials.

However, the general appearance of the tool is similar to that which is obtained during the machining of other aluminium alloys, and even the wear area, which manifests adhesion behavior that appears in the machining of other aluminium alloys in other processes [15,36]. Then, it is possible that the mechanism of predominant wear is secondary adhesion, as in most other commonly used aluminium alloys. However, some SiC particles have been deposited on the rake face. These particles can behave like the intermetallic particles in aluminium alloys, but with an increased abrasion power and concentration, and this increases abrasion and flank wear. This abrasion effect is different to all other aluminium alloys and provokes the rapid damage that has been shown previously.

**Figure 10:**
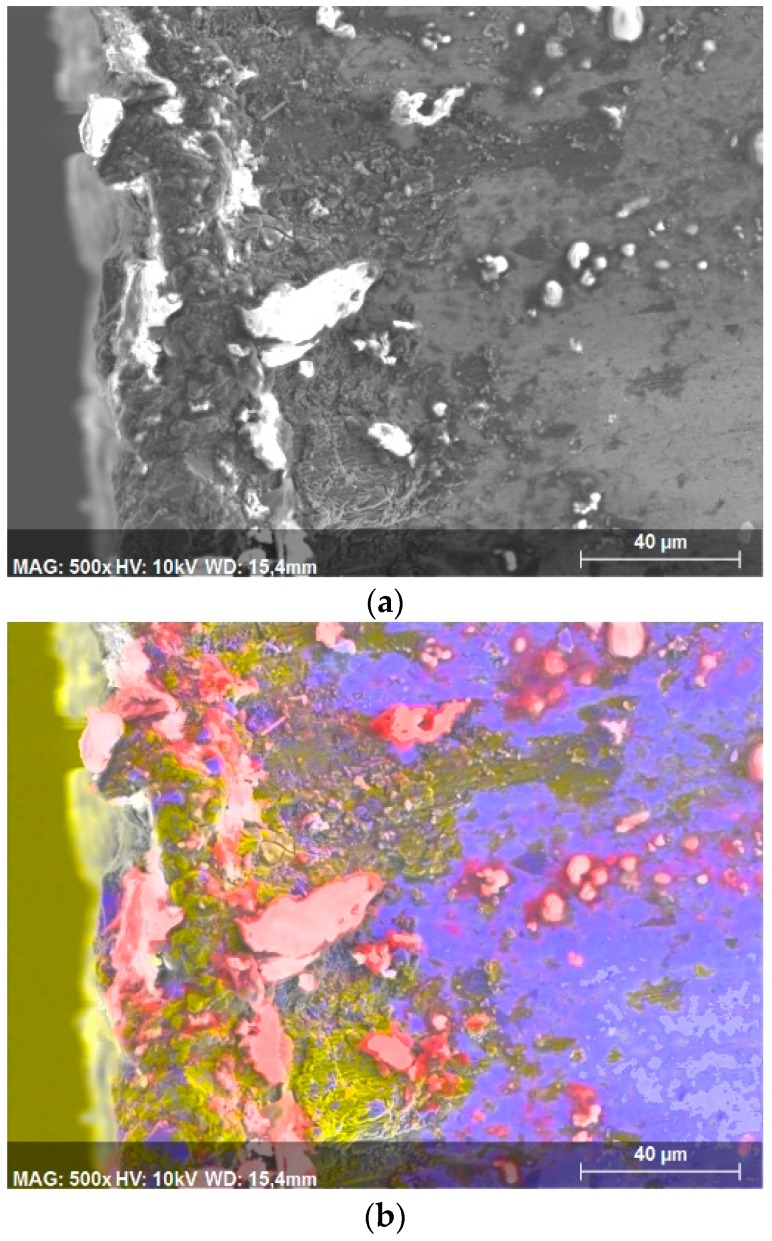
Scanning electron microscope analysis of the tool, where Vc = 40 m/min and d = 0.2 mm, and one tool path was used: (**a**) Original image. (**b**) Spectroscopy image with concentration by color: Al (yellow), W (blue), and Si (red).

The SiC particles were deposited close to the cutting edge (Figure 10), and although the rake face (blue area, tungsten rich area) was predominantly covered by aluminium (yellow area, aluminium-rich area), it appears that many areas are covered with material from the reinforcement (red area, carbon-rich area).

**Figure 11:**
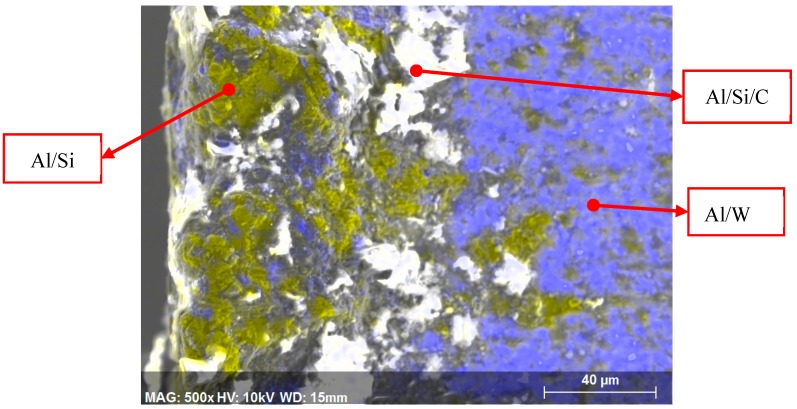
Spectroscopy image of compositional analysis of the cutting edge and the rake face (Vc = 20 m/min, d = 0.2 mm, one tool path).

Additionally, data taken from the characteristic zones (Figure 11) in the cutting edge show a high concentration of aluminium, which decreases when moving away from the edge (Figure 11, yellow areas). On the other hand, here, the reinforcements are distributed randomly, resulting in a non-homogeneous distribution of compounds. There are areas of very thin, electrotransparent material covered by the matrix alloy, whereas other areas only appear to be embedded with the reinforcement, as shown in the analysis earlier. It can be said that these reinforcements are firmly fixed on the tool without requiring the intervention of the aluminium (Figure 11, white zones).

Meanwhile, in areas close to the cutting edge, areas of both the matrix and reinforcement coexist, as has been previously seen. This suggests that although the secondary adhesion in the case of aluminium alloys is a thermomechanical process, in this material, the reinforcement induces a significant mechanical effect and, therefore, introduces the well-defined abrasion behavior.

**Figure 12:**
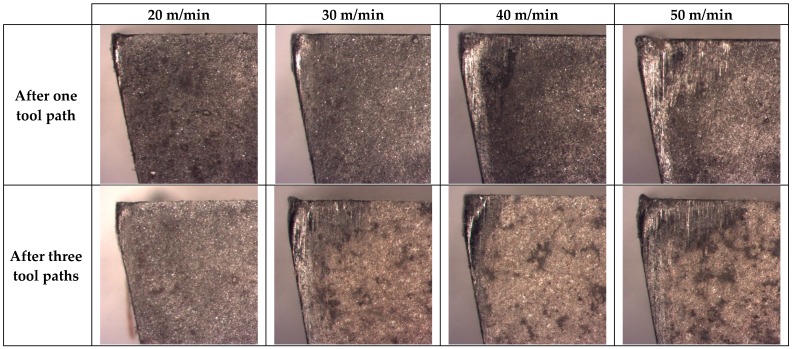
SOM images of the profile of the tool, where d = 0.2 mm in all of the cutting speeds studied.

To observe the flank wear, images of the profiles of the tools were analyzed (Figure 12). It is still possible to identify the material adhered and a high level of abrasion on the tools, denoted by the blurring observed in the high-speed film.

It is possible to appreciate a regrown edge in all tools, where bigger sizes arise from those produced at a higher cutting speed. This occurs similarly in other aluminium alloys during analogous processes [27,51]. Furthermore, it is possible to observe the effect of material loss in the tool, as well as flank wear. This damage is increased with the increase of cutting speed and time spent machining, and it also appears after short machining times.

This might indicate that the interval of stable evolution in machining is practically nonexistent, and the loss of material from the tool is produced in such a short time that it would lead to the catastrophic breakage of the tool after only very short machining times.

**Figure 13:**
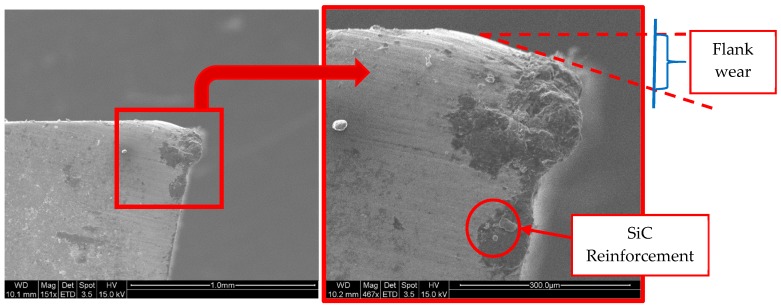
SEM analysis of the tool profile (Vc = 50 m/min, d = 0.2 mm, and three tool paths).

After analyzing the tool with SEM (Figure 13), it is possible to see a similar behavior with abrasive particles which are stuck or embedded on different areas of the tool, and the presence of lines of abrasion marks, symptomatic of flank wear, which, in this case, differs from the previous one in intensity, since it seems to be more pronounced. This behavior is maintained almost independently of machining time, although the loss of material of the tool is very marked in the first moments of machining and progresses rapidly. Therefore, abrasion is a very important phenomenon, in addition to (or possibly even more than) the secondary adhesion, which can lead to the futility of the tool after only a very short time.

**Figure 14:**
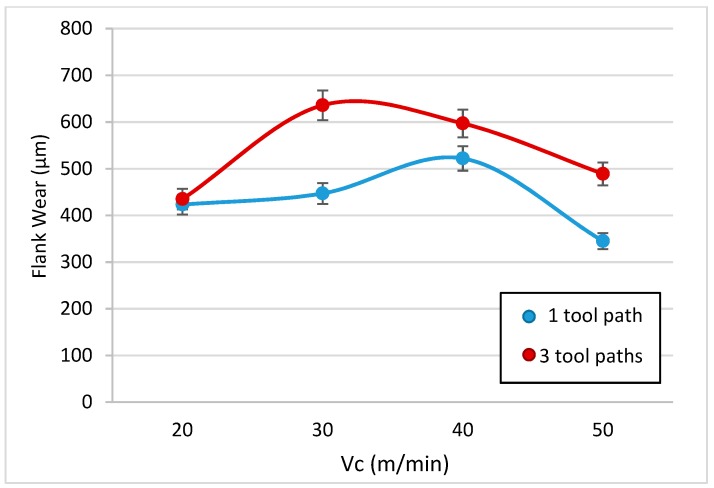
Flank wear evolution as a function of cutting speed and different machining times.

To analyze the loss of material in the flank, the profiles of the tools have been studied. The flank wear was measured using image processing techniques. The angle that forms the profile of the original rake face with the rake face after machining has been studied, in order to subsequently measure the parameter of flank wear according to ISO 3685. The data are shown in Table 5 and are represented in Figure 14.

It can be seen that there does not seem to be a definite pattern here, and this can be related with the random distribution of the reinforcement when it is projected. However, the flank wear in all cases was greater than the flank wear accepted by ISO 3685 (300 μm), and this value is exceeded after only very short machining times.

According to this value, the tool must be replaced after only one tool path. However, after three tool paths, a striking effect takes place; however, wear increases clearly in a few cases, which would lead to an increase in the adhered material, whereas in other cases, it appears to decrease. This manifested adhesion fills the gaps that were occupied by the tool material and, therefore, this apparently causes the value to drop.

This effect is false; however, as the bonded material does not display the mechanical properties of the tool, it will tend to fall off easily, pulling particles from the tool with it.

Therefore, the general trend is an increase in the loss of material from the tool in line with the increase in cutting speed and machining time, reaching values that exceed double the value recommended for the replacement of the tool after only a very short period of machining.

This flank wear provokes a tapping effect on the tool that increases the friction and affects the cutting forces. The values of the cutting forces in the different tests are shown in Table 6.

### 4.5. Cutting Forces

The machining of MMC reinforced with SiC generate chips of a “sawtooth” type, and thus a friction phenomenon appears, which causes fluctuation cutting forces [49]. The segmentation frequency of the sawtooth chips and the fluctuation frequency of the cutting forces are highly similar. This indicates that the formation of the sawtooth chips is the most important factor influencing the periodical fluctuation of the cutting force components in the turning of Inconel 718 [48].

In the traditional cutting process, the component of force in the direction of cutting, Fc, is the most important property of the component, and it has the biggest value. The second largest component, the vertical component of the force, Fy, is greatly different. Studying the data obtained, this can be corroborated; however, Fy is much higher than the expected values, being greater than the values of Fc even after three tool paths. Increasing the cutting speed and the machining time increases the cutting forces. This is the same behavior observed during the tool wear study.

**Figure 15:**
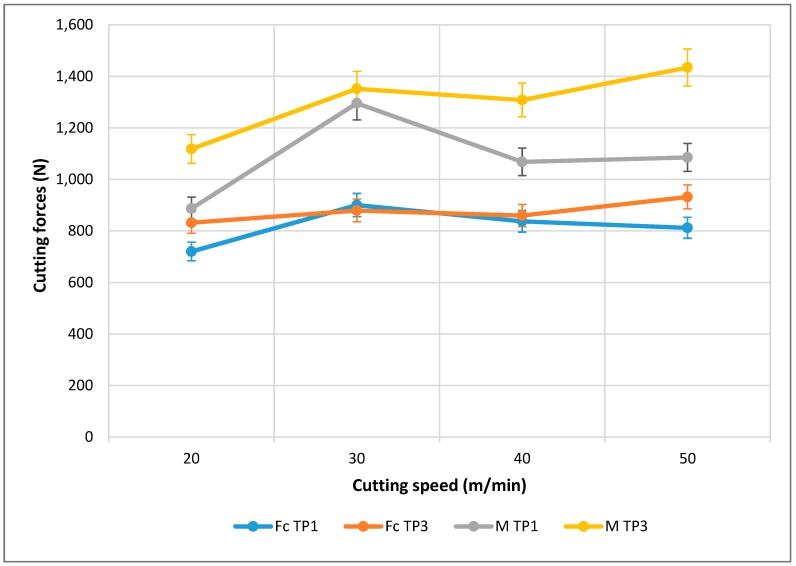
Cutting forces depending on the speed of cutting for several machining tool paths.

The forces registered in two axes have been combined to form resultant M; a comparative table; Table 6; and a graphic design, Figure 15.

According to the composition of the resultant of the forces, the modulus has been obtained through the square root of the quadratic addition of the components, as shown in the formula below.

$$M=\;\sqrt{Fc^{2}+Fy^{2}}$$

If analyzed more thoroughly (Figure 15), when considering only one tool path, it can be seen that with a Vc of 20, the values for both forces (Fc and M) are at their lowest. At this initial point, both values are increasing, where M is more significant than Fc, until reaching a peak Vc of 30, where both forces decreased until they were almost parallel up until a Vc of 40. Once this point was reached, from here to the end (Vc of 50) the values remained almost horizontal.

In the case of three tool paths, this is similar to the previous example. From Vcs of 20 to 30, the values increased; however, once reaching this peak, as in the case of one tool path, the values reduced until a Vc of 30. Between a Vc of 40 and 50, once again these values increased instead of remaining similar, as in the case of the previously registered values at a Vc of 40 for both cases, where Fc and M were both higher in the case of TP3 than TP1.

Even though the force Fc is similar in the one tool path case, the three tool path values are quite different for Fc or M, where M is always greater.

Note that although machining times are very low, this still provides evidence of the great influence of wear. This influence, as seen in the analysis of the tool, gives rise to the increase in cutting speed, which may cause increased wear and even an increase in flank wear. This would mean that the phenomenon of abrasion is increasingly more important, and, as noted, would lead to the catastrophic failure of the tool.

As seen in Figure 15, there is a dependency with the machining time that grows with the cutting speed. Slower speeds show an almost stabilized behavior, whereas the tendency is much more pronounced with increasing speeds.

On the other hand, in the case of the increased abrasion in the previously observed profile of the tool, which grows with the cutting speed, the phenomena causing this is lateral stress, and it therefore cannot be explained with standard theories about orthogonal cutting, thus an Fx force cannot exist.

When comparing the values of forces obtained with other light alloys, there are important differences to note. If compared with a Ti alloy [52], it can be seen that the forces are slightly lower when moving to depths of 0.2 mm in the range of 800 to 1500 N. However, when compared with forces that are obtained in an alloy of aluminium, it can be seen how the ranges are inferior and even with more aggressive settings and in more complex processes, they are only able to reach about 500 N [53]. This again confirms the hypothesis that increasing the concentration of reinforcement translates into increased tenacity, which causes an increase in the forces that by their inherent abrasive behavior leads to a more significant increase in damage, which is evident when considering the rapid loss of material in the cutting tools.

A brief summary of results can be found below.

- Microhardness has been studied here. The results show a wide range of values. The highest value belongs to the reinforcement material, whereas the lowest value belongs to the Al matrix. Intermediate values were obtained from matrix or interface areas with matrix reinforcement.
- Sawtooth chips were formed during machining. Some alloys employed in aeronautics, such as the titanium alloy Ti6Al4V or the nickel alloy known as Inconel 718, produce a similar behavior to that observed here, in terms of the chip formation mechanism and the type of chip resulting from the machining.
- Cyclic variation of the shear angle along the machining process can be noticed. Attending to the ISO 3685:1993 standard, the chips formed during the cutting process are of the 6.2 type. The addition of SiC particle reinforcement into the aluminium matrix caused a reduction in its ductility and makes the material ideal for producing semicontinuous chips.
- Tool wear is significant in all cases. Here, secondary adhesion appears (characteristic of Al alloys). The main factor of abrasion wear is the SiC reinforcement, which induces flank wear. A clear relationship with the cutting parameters has not been revealed. The Shaffer model could not be applied to these materials, as the values obtained differ by more than 80% of the calculated value.
- It is possible to appreciate a regrown edge in all tools, where a bigger size is found for those tools produced at higher cutting speeds. Furthermore, it is possible to observe the effect of material loss in the tool, as well as flank wear. This damage is increased with the cutting speed and the time spent machining, and it appears even after short machining times.
- The cutting force Fy showed much higher values than expected, where even after three tool paths it was above the value of Fc. Even though the force Fc is relatively similar between the one and three tool path cases, the values of Fc and M are quite different, where M is always greater than Fc.

## 5. Conclusions

Regarding the chip analysis, this confirmed the behavior previously described by other authors, showing a shredded arch type with a segmented or sawtooth morphology.

The tools have suffered severe wear, even in the best cutting conditions.

With regard to the cutting forces, it can be ensured that the effect of abrasion causes a predominant effect of friction on the rake face that manifests itself in a very marked increase in the force Fy.

On the other hand, the machining time is a determining factor and the increase in the wear causes a simultaneous increase in the forces, reaching values close to those found in alloys in less than one second, and surpassing those of most other aluminium alloys.

The best results have been obtained with the average values of the parameters: speed and deep of cut. The highest speed and lowest depth of cut is not always the best option.

## Figures and Tables

**Table 1:** Composition of the metallic matrix composite (MMC) matrix alloy [38].

Alloy	Al	Cu	Fe	Mg	Mn	Si	Ti	Zn	Others
UNS Al356.2	91.30–93.20	0.10	0.12	0.30–0.45	0.05	6.5–7.5	0.20	0.05	0.15
A356.2	91.10–93.30	≤0.20	≤0.20	0.25–0.45	≤0.10	6.5–7.5	≤0.20	≤0.10	0.15

**Table 2:** Composition and some properties of the MMC used [39].

Composition and Properties	AlSiC-9
Matrix: Aluminium alloy A 356.2	37 vol %
Reinforcement: SiC	63 vol %
Density (g/cm^3^)	3.01
Thermal conductivity (W/K∙m) at 298.15 K	190
Specific heat (J/g∙K) at 298.15 K	0.741
Young’s modulus (GPa)	188
Coulomb, shear modulus (GPa)	76

**Table 3:** Machining parameters MMC AlSiC.

**Cutting Speed (Vc)**	20, 30, 40, 50 m/min
**Depth of Cut (DoC)**	0.10, 0.20 mm

**Table 4:** Microhardness (HV) of the aluminium MMC.

Sample	Force (N)	Time (seconds)	HV (kg/mm^2^)	Notes
1	0.98	15	1786	Measured in reinforcement
2	1.96	15	262	Measured in matrix
3	1.96	15	600	Measured in interface
4	1.96	15	186	Measured in matrix

**Table 5:** Values of angle wear and flank wear according to the cutting parameters.

	Machined Length	20 m/min	30 m/min	40 m/min	50 m/min
Angle wear (°)	one tool path	7.85	7.72	13.94	5.28
	three tool paths	2.56	12.12	3.95	14.39
Flank wear (μm)	one tool path	423.01	447.00	522.01	345.01
	three tool paths	435.00	636.00	597.01	489.00

**Table 6:** Average values of the cutting forces obtained in the machining tests.

Vc	Machined Length	Fc (N)	M (N)
20 m/min	one tool path	720,000	887,010
	three tool paths	832,007	1,118,255
30 m/min	one tool path	900,277	1,295,552
	three tool paths	879,159	1,351,942
40 m/min	one tool path	837,356	1,068,034
	three tool paths	859,668	1,308,143
50 m/min	one tool path	811,942	1,085,266
	three tool paths	931,908	1,434,406
